# Depressive symptoms of female nursing staff working in stressful environments and their association with serum creatine kinase and lactate dehydrogenase – a preliminary study

**DOI:** 10.1186/1751-0759-8-21

**Published:** 2014-09-09

**Authors:** Ayako Kato, Hiroyuki Sakakibara, Hirohito Tsuboi, Asami Tatsumi, Masanobu Akimoto, Kayoko Shimoi, Takeshi Ishii, Hiroshi Kaneko, Tsutomu Nakayama, Norio Ohashi

**Affiliations:** 1 Department of Food and Nutritional Sciences, Graduate School of Integrated Pharmaceutical and Nutritional Sciences, University of Shizuoka, 52-1 Yada, Suruga-ku, Shizuoka, 422–8526, Japan; 2 Prima Meat Packers, Ltd., Tsuchiura, Japan; 3 Department of Agriculture, University of Miyazaki, Miyazaki, Japan; 4 Department of Neurology and Psychosomatic Medicine, Bantane Hospital, Fujita Health University School of Medicine, Nagoya, Japan; 5 Institute of Medical, Pharmaceutical & Health Sciences, Kanazawa University, Kanazawa, Japan; 6 Faculty of Nursing, Hamamatsu University School of Medicine, Hamamatsu, Japan; 7 Faculty of Applied Life Science, Nippon Veterinary and Life Science University, Musashino, Japan

**Keywords:** Creatine kinase, Center for Epidemiologic Studies Depression scale, Depressive symptom, Healthy female nursing staff

## Abstract

**Background:**

The activity of creatine kinase (CK) in serum has recently been reported to be potentially associated with several types of depression. The aim of this study is to evaluate whether serum enzymes, including CK, vary even in a healthy population with depressive symptoms caused by work-related stress. We gave questionnaires and blood examinations to 93 healthy female nursing home workers and did an enzyme-linked immunosorbent assay for the quantitative detection of CK isozyme muscle-type M chain (CK-MM) in serum.

**Findings:**

Depressive symptoms were determined using the Center for Epidemiologic Studies Depression (CES-D) scale and compared with the results of the blood examination and serum CK-MM levels. The CES-D results showed significant negative correlations with total CK and lactate dehydrogenase (LDH) activities and CK-MM level (*r* = -0.29, *p* = 0.0062; *r* = -0.29, *p* = 0.0065; *r* = -0.33, *p* = 0.0016, respectively).

**Conclusions:**

Total CK and LDH activities and serum CK-MM level appear to be associated with the depressive symptoms of healthy nurses working in stressful environments, although the significance level was relatively low. The simultaneous detection of serum CK and LDH activities or serum CK-MM level and LDH activity may be useful as an indicator of depressive symptoms, at least for female nursing staff with work-related stress.

## Findings

Several professions, such as nursing and teaching, are reported to have a working environment with frequent exposure to stressful events [1,2]. Such daily work-related stress is recognized as one of the important causes of adverse symptoms such as depression or depressive symptoms [3]. In humans, depression is often comorbid with other chronic diseases and can worsen their associated health outcomes [4]. Previous studies have suggested that the activities of serum enzymes such as creatine kinase (CK) are potentially involved in several types of depression [5,6]. Segal et al. investigated the total activity of serum CK in various forms of depression, including major depression with or without psychotic symptoms, bipolar depression, and schizoaffective depression [5]. Their results showed that serum CK activity in nonpsychotic major depression was significantly higher than that in all other forms of depression. Additionally, Feier and colleagues reported that the serum CK activity of bipolar depression patients was higher than that of healthy control subjects and depressed patients [6]. CK is an enzyme that catalyzes the reversible phosphorylation of ATP and creatine to ADP and phosphocreatine, and it plays an important role in the regulation and maintenance of cellular energy metabolism [7,8]. CK is usually categorized into three isozymes consisting of combinations of muscle (M) and brain (B) subunits: CK-BB, the brain type; CK-MM, the muscle type; and CK-MB, the cardiac type [8]. It is well known that blood contains predominantly the CK-MM isozyme, followed by CK-MB, but not CK-BB. This previous research led us to hypothesize that serum CK activity, especially that of CK-MM and/or other potential serum enzymes derived from muscle, might be associated with depressive symptoms, even in healthy subjects with work-related stress. Therefore, this study investigated the correlation of depressive symptoms of nursing staff on active duty in stressful environments with total CK activity, serum CK-MM level, and other serum enzymes.

The participants were 93 healthy female healthcare staff (mean age: 40.1 ± 13.4 [mean ± SD]) in two nursing homes located in Shizuoka prefecture, Japan. The depressive symptoms were evaluated using the Center for Epidemiologic Studies Depression (CES-D) scale [9]. On the first day after the questionnaire (including items on daily habits such as smoking, alcohol consumption, and leisure-time physical activities) was self-administered, blood samples were collected in the morning (8:00 am to 9:00 am) after fasting overnight. The study protocol was approved by the Ethics Committee of the University of Shizuoka, which confirmed that the study design was in accordance with the Declaration of Helsinki. Written informed consent was obtained after a complete description of the study to each participant.

The serum and EDTA-treated plasma were separated by centrifugation at 1,500 × g for 20 min, and delivered to FALCO Biosystems Ltd. (Kyoto, Japan) for the determination of biochemical parameters such as total CK, aspartate aminotransferase (AST), alanine aminotransferase (ALT) and lactate dehydrogenase (LDH) activities and blood urea nitrogen (BUN) and cortisol levels. Body mass index (BMI) was calculated from body weight and height. Serum CK-MM levels were quantified by an enzyme-linked immunosorbent assay using the method described in our previous report [10].

The Japanese version of IBM SPSS Statistics software for Mac OS (ver. 19) was used for statistical analyses. Correlations among each variable were calculated, controlling for age, BMI, smoking (non-smoker *vs* ex- or present smoker); alcohol consumption (once or less per week *vs* twice or more per week); and exercise (once or less per month *vs* twice or more per month). Total CK activity, CK-MM level, and AST and ALT activities were logarithmized for the correlation analyses. *P* values less than 0.05 were considered significant.

**Figure 1:**
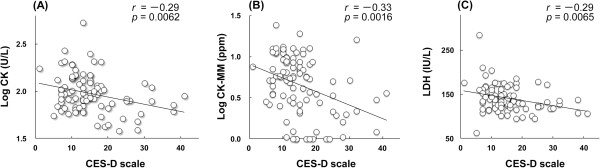
**Correlations between the CES-D scale and blood components.** Correlations between CES-D scale and **(A)** total CK activity, **(B)** CK-MM level, and **(C)** LDH activity in serum. Values are controlled for age, BMI, smoking habit, alcohol consumption, and leisure-time-physical activities.

The characteristics of the participants are summarized in Table 1. The results indicate that the participants were physically healthy on the basis of biochemical parameters, as the respective parameter levels were within the normal ranges recommended by the Japanese Ministry of Health Labour and Welfare. The CES-D scale was obtained by the questionnaire, and a higher score indicated potentially significant levels of depressive symptomatology [9]. Table 2 displays the correlations among each variable after adjusting for age, BMI, smoking, alcohol consumption, and physical activity. As shown in Table 2 and Figure 1, the total CK activity of the participants showed significant negative correlations with depressive symptoms evaluated by CES-D scale (*r* = -0.29, *p* = 0.0062). The more depressive subjects showed significantly less total serum CK activity. The CK-MM levels, which are likely responsible for the majority of serum CK activity, also showed a significant negative correlation with depressive symptoms evaluated by CES-D (*r* = -0.33, *p* = 0.0016). Additionally, we found that LDH activity in sera from participants showed a significant negative correlation with depressive symptoms evaluated by CES-D (*r* = -0.29, *p* = 0.0065). The negative correlations in the LDH activity were similar to those in total CK activity and CK-MM levels in serum, as described above. The enzyme activities of AST and ALT and the BUN level in sera showed no relationship with depressive symptoms as determined by CES-D (Table 2). Furthermore, the serum cortisol level, which is commonly used as a stress indicator [11], showed no correlation with CES-D-determined depressive symptoms in the present study, most likely due to diurnal variations in the cortisol level [12,13].

**Table 1:** Participant characteristics (n = 93)

		**Mean (SD)**
**Demographic data**	Age	40.1 (13.4)
	BMI (kg/m^2^)	22.8 (4.24)
**Serum data**	Cortisol (μg/dL)	11.5 (5.43)
	CK (U/L)	106 (65.2)
	CK-MM (ppm)	5.08 (4.49)
	LDH (IU/L)	141 (30.2)
	BUN (mg/dL)	13.3 (3.92)
	AST (IU/L)	20.3 (7.31)
	ALT (IU/L)	18.3 (14.6)
		**n**
**Smoking habit**	Non smokers	53
	Ex- and present smokers	40
**Alcohol consumption**	Once or less than once per week	72
	More than twice per week	11
**Leisure-time physical activities**	Once or less than once per month	62
	More than twice per month	31

ALT, alanine aminotransferase; AST, aspartate aminotransferase; BUN, blood urea nitrogen; CK, creatine kinase; CK-MM, creatine kinase isozyme muscle M chain; LDH, lactate dehydrogenase.

**Table 2:** Correlations among depressive symptoms and biochemical values

	**CES-D scale**	**Cortisol**	**Log CK**	**Log CK-MM**	**LDH**	**BUN**	**Log AST**	**Log ALT**	**AST/ALT ratio**
CES-D scale		0.084	-0.29^**^	-0.33^***^	-0.29^**^	0.002	0.047	0.12	-0.14
Cortisol			0.013	-0.21^*^	-0.022	0.12	0.094	0.088	0.013
Log CK				0.59^****^	0.17	0.15	0.31^***^	0.15	0.11
Log CK-MM					0.26^*^	0.067	0.16	0.11	-0.038
LDH						0.076	0.17	0.13	-0.035
BUN							0.34^***^	0.39^****^	-0.23^*^
Log AST								0.79^****^	-0.17
Log ALT									-0.72
AST/ALT ratio									

Correlations are controlled for age, BMI, smoking habit, alcohol consumption, and leisure-time physical activities.

ALT, alanine aminotransferase; AST, aspartate aminotransferase; BUN, blood urea nitrogen; CK, creatine kinase; CK-MM, creatine kinase isozyme muscle M chain; LDH, lactate dehydrogenase.

*p < 0.05, **p < 0.01, ***p < 0.005, ****p < 0.001.

Several previous studies have suggested that serum enzymes related to muscle origin, such as CK or LDH, appear to be associated with psychiatric illnesses, including mania, depression, and bipolar disorder [5,6,14,15]. In particular, mania appears to induce the release of CK and LDH from skeletal muscle, occasionally at unusually high levels, and these enzymes are presumably released from skeletal muscle in association with agitation, muscle tension, or blood stasis and local tissue hypoxia consequent to hypoactivity [14]. Segal et al. have shown that serum CK activity in nonpsychotic major depression was significantly higher than that in psychotic major depression, bipolar depression, and schizoaffective depression [5]. In bipolar disorder, Segal et al. have also shown that CK activity was higher in the manic phase than in the depressive phase, suggesting that clinical differences between the manic and depressive states are supported by contrasting levels of CK [15]. Additionally, Feier et al. have similarly shown that serum CK activity in the manic phase was higher than that in the depressive phase of bipolar disorder and that it was also higher than activity in healthy volunteer controls [6]. In the present study, the depressive symptoms evaluated by CES-D were also negatively correlated not only with the serum enzyme activities of muscle-related CK and LDH, but also with the protein levels of serum CK-MM, even in physiological healthy nursing staff working in conditions that produced work-related stress. Other serum enzymes and biochemical parameters that are less related to muscle origin, such as AST, did not appear to be correlated with depressive symptoms.

In conclusion, we found that total CK and LDH activities and the protein level of CK-MM in serum (muscle-related enzymes) appear to be associated with the depressive symptoms of healthy active duty nurses working under the stressful conditions, although the significance level was relatively low. The simultaneous detection of serum CK and LDH activities or serum CK-MM level and LDH activity may be useful as indicators of depressive symptoms and also as objective indices of the health of working environments, at least for female nursing staff with potential work-related stress, although the causal association of these factors remains uncertain.

## Abbreviations

CES-D: Center for Epidemiologic Studies Depression; CK: Creatine kinase; CK-MM: Creatine kinase isozyme muscle M chain; LDH: Lactate dehydrogenase; ALT: Alanine aminotransferase; AST: Aspartate aminotransferase; BUN: Blood urea nitrogen.

## Competing interests

The authors declare that they have no competing interests.

## Authors’ contributions

AK carried out the immunoassays and drafted the manuscript. HS helped with the human study, data collection and drafting of the manuscript. HT mainly designed the human study, carried out data collection and statistical analysis, and drafted the manuscript. AT and HK designed and helped with the human study. MA carried out the immunoassays. KS mainly designed the human study and carried out the data collection. TI and TN participated in the design and coordination of the study. NO organized the whole study and drafted the manuscript. All authors have read and approved the final manuscript.
